# Metformin, aging and cancer

**DOI:** 10.18632/aging.100556

**Published:** 2013-05-09

**Authors:** Olga Moiseeva, Xavier-Deschênes-Simard, Michael Pollak, Gerardo Ferbeyre

**Affiliations:** Département de Biochimie, Université de Montréal, Montréal, Québec H3C 3J7, Canada

Many cancers are associated with aging [1]. Metformin, a widely used antidiabetic drug, has been linked to a reduced cancer incidence in some retrospective, hypothesis-generating studies [2]. Since cancer and aging may share certain molecular processes, it is plausible that metformin may prevent cancer by acting on the aging process. Consistent with this idea, several studies report a life span extension in animal models after treatment with metformin [3].

What is the mechanism by which aging may increase cancer incidence? Although many molecular changes correlate with aging, the presence of senescent cells capable of secreting inflammatory cytokines may be involved. This senescence associated secretory phenotype (SASP) consists of multiple cytokines, chemokines, growth factors and extracellular matrix degrading enzymes that can potentially affect normal tissue structure [4]. The SASP probably evolved as a gene expression program to assist the senescent tumor suppression response and tissue repair after damage and should be viewed as an initial adaptive response [5]. However, like acute inflammation, the SASP should be turned off to avoid maladaptive consequences. In some contexts, senescent cells are cleared by professional phagocytic cells [6] and this mechanism avoids any further complications. On the other hand, if senescent cells escape clearance, mechanisms that prevent the SASP should operate to avoid chronic inflammation and tissue disruption. Such endogenous mechanisms for clearing senescent cells or suppressing the SASP may fail with age. As a consequence, chronic SASP may cause a microenvironment in old tissues that facilitates tumor initiation and then stimulates cancer cell growth, motility and angiogenic activity. This unfortunate interaction between senescent cells and cancer cells has been reproduced in experimental mouse models where senescent fibroblasts stimulated tumor progression [4]). The mechanisms of senescent cell clearance and SASP control are not yet known. However, during experiments to study the potential cancer prevention activity of metformin, we found serendipitously that the drug prevented the expression of many proteases, cytokines and chemokines in senescent cells [7].

**Figure 1:**
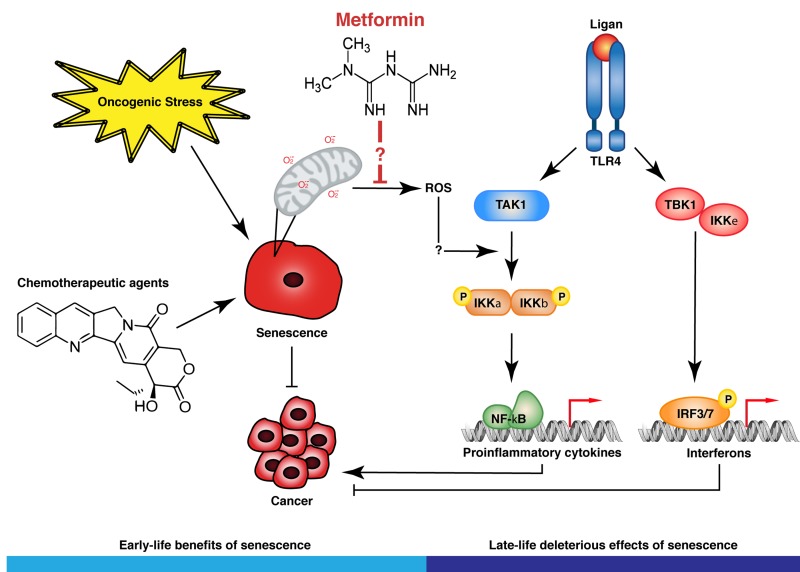
Metformin inhibits the activation of IKK kinases in senescent cells The model proposes that metformin reduces ROS generation by mitochondria preventing the activation of IKK kinases a step that is ROS-sensitive. Metformin does not affect the activation of the interferon response in senescent cells suggesting that it modulates the senescence associated secretory phenotype in a way that reduces chronic inflammation but not tumor suppression.

At the molecular level, we found that metformin interfered with the activation of protein kinases IKK a and b, which are responsible for activating NF-kB, an essential transcription factor for SASP activation. Intriguingly, metformin did not reduce the expression of anticancer cytokines such as interferon and interferon target genes in senescent cells, suggesting that it modulates SASP to reduce its inflammatory potential but retaining its antitumor activity. In addition, metformin did not affect the senescent cell cycle arrest caused by oncogenic ras in primary human cells, suggesting again that it can modulate the SASP without allowing proliferation of potentially malignant cells. The primary site of action of metformin is considered to be the complex I of the electron transport chain [2]. However, molecular details of the interaction between metformin and complex I remain to be identified. Complex I is one of the main cellular sources for reactive oxygen species (ROS) and we have shown that metformin can prevent ROS production by senescent cells [8]. It is thus plausible that ROS links senescence to NF-kB activation and that metformin interferes with this mechanism by acting on complex I (Fig 1). Metformin is not immunosuppressive so its ability to inhibit NF-kB is likely confined to certain pro-inflammatory contexts such as senescence. We thus propose that metformin prevents cancer by modulating the SASP in tissues where senescent cells were not naturally cleared.

Many questions remain to be addressed in order to fully characterize metformin actions. Our results were obtained using cultured senescent fibroblasts and macrophages; other cell types should be studied as well. In addition, it remains to be determined if metformin can achieve this anti-SASP activity in vivo or whether it can influence the clearance of senescent cells by modulating the SASP. Anisimov and colleagues reported that metformin extends life span in female mice but not males [3] and it would be interesting to study whether NF-kB and SASP inhibition by metformin is gender dependent. Additional epidemiological data and laboratory experiments may justify well-designed clinical studies to evaluate metformin as a cancer preventive agent in specific contexts where its recently described actions would be hypothesized to be useful.
